# COVID-19-Associated Miller Fisher Syndrome With Long Latency Period: A Case Report

**DOI:** 10.7759/cureus.24638

**Published:** 2022-05-01

**Authors:** Wayne Kuang, Priya Desai, Alexander Voloshko, Deepthi Jayasekara

**Affiliations:** 1 Pediatrics, Los Angeles County+University of Southern California Medical Center, Los Angeles, USA; 2 Internal Medicine, Western University of Health Sciences, Pomona, USA; 3 Internal Medicine, Scripps Mercy Hospital, San Diego, USA; 4 Internal Medicine, Santa Barbara Cottage Hospital, Santa Barbara, USA; 5 Infectious Disease, Emanate Health Queen of the Valley Hospital, West Covina, USA

**Keywords:** guillain-barrè syndrome, late-onset, young adult male, miller-fisher syndrome, covid-19

## Abstract

Coronavirus disease 2019 (COVID-19) has been known to affect multiple organ systems, aside from the respiratory system. Increasing reports of post-infection neurological complications have been reported. Miller-Fisher syndrome, a rare variant of Guillain-Barré syndrome (GBS), has been reported after COVID-19 infection. We present the youngest known adult (26-year-old male) reported to have had COVID-19-associated Miller Fisher syndrome (MFS) with also the longest reported latency period (10 weeks) between infection and development of neurological symptoms (including dysphagia, horizontal diplopia, facial weakness, upper and lower extremity weakness, paresthesia). This is currently the second youngest reported case after the case of a seven-year-old child. The patient was treated with intravenous immunoglobulin and was ultimately transferred to a different facility for higher level of care. Most symptoms were resolved within four days. The patient reported resolution of neurologic symptoms with the exception of left-sided facial weakness at the one-year follow-up. As more reports of COVID-19-associated GBS and MFS appear in the future, we are likely to discover more variability than was previously known in GBS and MFS. With COVID-19 potentially affecting multiple systems, there could be increased variability to previously known conditions. Future studies may need to closely examine long-term follow-ups of patients previously diagnosed with COVID-19 as post-COVID complications become more prevalent.

## Introduction

Coronavirus disease 2019 (COVID-19) is an infectious disease caused by the severe acute respiratory syndrome coronavirus 2 (SARS-CoV-2). Common symptoms of COVID-19 include shortness of breath, non-productive cough, fatigue, and fever, demonstrating that SARS-CoV-2 primarily affects the respiratory system and can result in acute respiratory illness [1].

There have been numerous reports of symptoms outside the respiratory system, including ageusia and anosmia, diarrhea, encephalopathy, and cerebrovascular disease, which indicates that SARS-CoV-2 is capable of affecting multiple organ systems [2-4]. In a mini-review, various presentations of COVID-19 were noticed to even occur before the development of respiratory symptoms and thus clinicians should be aware of these to facilitate diagnosis and management [5].

COVID-19 has been around now for two years, and despite having reliable testing methods, there are various post-COVID complications. It has been well established that acute COVID-19 infection can lead to neurologic sequelae [2]. Recently, there have been reports of Miller Fisher syndrome (MFS) in patients with COVID-19 [6]. MFS is a rare variant of Guillain-Barré syndrome (GBS), an autoimmune condition that encompasses a variety of different immune-mediated polyneuropathies [7]. Unlike the bilateral ascending paralysis characteristic of the most common GBS subtype, acute inflammatory demyelinating polyneuropathy (AIDP), MFS typically presents as ophthalmoplegia with ataxia and areflexia [8]. Involvement of the third, fourth, and sixth cranial nerves is typical of MFS; however, the syndrome can also involve other cranial nerves as well as peripheral nerves, resulting in symptoms such as facial, bulbar, and extremity weakness [9]. Like other GBS variants, MFS is believed to be caused by a maladaptive response to infection, with a viral infection preceding the condition in 72% of cases, by a period of 10 days on average [9]. This case report discussed the presentation of a 26-year-old male diagnosed with MFS, which was preceded by a mild COVID-19 infection 10 weeks prior.

## Case presentation

A 26-year-old male presented to the emergency department with sudden onset right-sided weakness for one day, preceded by 10 days of worsening dysphagia and hoarseness, right facial droop, and numbness and tingling of the right face and tongue. The patient’s past medical history was significant for COVID-19 infection in September 2020, approximately 10 weeks prior to admission. Patient was unvaccinated since the vaccine was not available to the public at the time. Patient otherwise had no significant medical history, prescription medication history, family history, or social history. He reported mild symptoms including generalized weakness, loss of taste and smell, nausea, diarrhea, and myalgias for six days, not requiring hospitalization or treatment. At the time, he did not experience any respiratory symptoms from the COVID-19 infection.

The patient was slightly hypertensive on admission (blood pressure of 150/98), but otherwise hemodynamically stable. The right-sided weakness was progressive to numbness and tingling of bilateral fingers and toes, which spread proximally to his bilateral arms and legs. The progression of bilateral extremity weakness and paresthesia was followed by development of horizontal diplopia, left-sided facial weakness, and worsening dysphagia. He denied bowel or bladder symptoms.

Neurological examination revealed paresis of the upper and lower facial nerves, ptosis, weakness of the right genioglossus, dramatically weakened grip strength of the right side compared to left, hyporeflexia of the left upper extremity, areflexia of the right upper extremity and bilateral lower extremities, and mild dysarthria. He was found to be mildly ataxic with difficulty supporting weight on the right lower extremity. The patient demonstrated decreased sensation to light touch in a stocking-and-glove distribution bilaterally. Sensation to light touch of the face, cerebellar examination, and gag reflex were normal.

Complete blood count and metabolic panel were within normal limits on admission. The patient tested positive with the antinuclear antibodies (ANA) screen and the anti-Sjögren's-syndrome-related antigen A (anti-SSA) autoantibodies. Herpes simplex virus (HSV) I and II deoxyribonucleic acid (DNA) polymerase chain reaction (PCR) were not detected. Rapid plasma reagin (RPR) was non-reactive. Cerebrospinal fluid (CSF) Lyme antibody index screened negative. Severe acute respiratory syndrome coronavirus 2 (SARS-CoV-2) PCR was indeterminate at the time of admission. Gram stain and CSF culture showed no growth after five days. CSF analysis revealed albuminocytologic dissociation, with total protein of 54 mg/dL and white blood cell count of 0 cells/uL. Magnetic resonance imaging (MRI) of brain with and without contrast showed no abnormalities. MRI of cervical spine with and without contrast revealed multilevel neuroforaminal stenosis, including moderate-to-severe right neural foraminal stenosis at the levels of C3-C4, but no evidence of spinal canal stenosis or disc herniation. Computed tomography (CT) of soft tissue neck with contrast showed no evidence of underlying airway obstruction. Overall, the imagings showed no clinically significant findings.

The patient was admitted for further evaluation of right-sided weakness, suspicious for GBS, MFS variant. He was treated with the standard single intravenous immunoglobulin (IVIG) dose (0.4 g/kg body weight for five days), which was 35 g of IVIG for this patient. He was ultimately transferred to a different facility for higher level of care. The paresthesia and weakness of the right face and bilateral upper and lower extremities resolved within four days of initiation of IVIG treatment. The patient reported resolution of neurologic symptoms with the exception of left-sided facial weakness at one-year follow-up.

## Discussion

In this case, COVID-19-associated MFS was determined to be the most likely diagnosis after detailed history-taking and examining the lab results. There is a potential possibility that there was an additional viral cause aside from the SARS-CoV-2 infection; however, that possibility could not be determined from the history and from the lab results. Although 10 weeks latency period is unusually long, there is some evidence that about one-third of patients who develop GBS report symptoms of an infection that occurred over six weeks preceding the onset of the condition [10].

In many ways, the presentation of our patient’s neurological symptoms following his infection with SARS-CoV-2 is similar to that of several other previously documented cases of COVID-19 associated with MFS. While there were some cases of MFS seen following a severe COVID-19 infection requiring hospitalization [11], the majority of MFS cases documented occurred in patients with mild cases of COVID-19, much like our patient [6]. In some reports, patients with a positive COVID-19 test had presentations consistent with MFS despite never having symptomatic COVID-19, highlighting that the syndrome can potentially present in any patient exposed to SARS-CoV-2, regardless of disease severity [6].

Previous reports of COVID-19-associated MFS vary significantly in their clinical presentations, but the majority include the classic triad of ophthalmoplegia, ataxia, and areflexia, with various additional neurologic symptoms [6]. Our patient had clear ataxia and areflexia on examination but had no ophthalmoplegia. He did, however, have marked progressive facial weakness and numbness, indicating likely involvement of cranial nerves V and VII, a pattern also occasionally seen in MFS. The marked extremity weakness and numbness seen in our patient have also been previously documented in a COVID-19-associated MFS [12].

Our patient’s presentation demonstrates several significant differences in comparison to prior cases of COVID-19-associated MFS. Notably, our patient is the youngest adult documented case to date, at the age of 26 years, with the next youngest adult recorded to be a 36-year-old male [13]. Furthermore, our patient had no known relevant past medical history of predisposing conditions for neurologic complications from COVID-19. He reported no hypoxemia or other respiratory symptoms at the time of the COVID-19 infection. This is indicative that COVID-19-associated MFS is not limited by age and clinicians will likely see increased incidents in younger populations, not just in the older populations. In fact, there was a recent case report of a seven-year-old child, the youngest to be reported so far, who developed COVID-19-associated MFS [14].

This case also describes the longest latency period of 10 weeks between the onset of COVID-19 viral symptoms and the appearance of neurological symptoms, with the average latency period being 10 days [9]. The patient described continued to experience left-sided facial weakness at one-year follow-up in stark contrast to previous cases in which patients reported complete resolution of symptoms within days to weeks of treatment [15,16]. These are suggestive that as the prevalence of post-COVID complications increases, clinicians should be aware that complications may even arise months after the initial COVID infection.

A possible limitation of this study is the lack of anti-ganglioside Q1b (anti-GQ1b) antibody testing which is often used, but not essential, to help diagnose Miller Fisher syndrome. A review of 123 patients with MFS found that 85% were positive for anti-GQ1b [17]. However, a systematic review in 2021 found that a majority of COVID-19-associated MFS cases had negative anti-GQ1b results [6]. There is an additional possibility that the positive anti-GQ1b results came from non-COVID-19 infections. The anti-GQ1b association and the exact pathophysiology of COVID-19 infection leading to the demyelination of the peripheral nervous system are still unclear and warrant further research. Neurophysiological studies, specifically electromyography, were also not performed at our facility as the patient was transferred to a tertiary center shortly after admission.

Ultimately, our patient was treated with intravenous immunoglobulin, which led to a marked improvement in symptoms, with only mild left-sided facial weakness remaining. Our case reaffirms the effectiveness of IVIG treatments in COVID-19-associated MFS, though it is difficult to infer conclusions regarding the optimal dose.

## Conclusions

This case report describes the characteristics of COVID-19-associated Miller Fisher syndrome. Our patient, at the age of 26 years, is currently the youngest recorded adult case and the second youngest case after the case of a seven-year-old child. This case report has the longest latency period of 10 weeks post-COVID-19 infection before symptoms appeared, and is also the first to examine the clinical symptoms one year after treatment with IVIG. SARS-CoV-2 RNA was not detected in the CSF analyses when the patient presented with neurological symptoms. Patient exhibited a good clinical outcome after treatment with IVIG with only remnants of left-sided facial weakness at the one-year follow-up, indicative that IVIG is still an effective treatment for COVID-19 related MFS. As more reports of COVID-associated GBS and MFS appear in the future, we are likely to discover more variability than was previously known in GBS and MFS, likely due to the effects that COVID-19 has on multiple organ systems. We are also more likely to see additional pediatric cases in the future as well. The unclear and potential lack of association with anti-GQ1b and the unclear pathophysiology of COVID-19 infection leading to the demyelination of the peripheral nervous system warrants further research. Future studies should also consider closely examining long-term follow-ups of patients previously diagnosed with COVID-19 as post-COVID complications become more prevalent. Lastly, the long latency of this report indicates that clinicians should be aware that complications may even arise months after the initial COVID-19 infection.
